# The Influence of Tumor Microenvironment on Immune Escape of Melanoma

**DOI:** 10.3390/ijms21218359

**Published:** 2020-11-07

**Authors:** Aleksandra Simiczyjew, Ewelina Dratkiewicz, Justyna Mazurkiewicz, Marcin Ziętek, Rafał Matkowski, Dorota Nowak

**Affiliations:** 1 Department of Cell Pathology, Faculty of Biotechnology, University of Wroclaw, Joliot-Curie 14a, 50-383 Wroclaw, Poland; ewelina.dratkiewicz@uwr.edu.pl (E.D.); justyna.mazurkiewicz2@uwr.edu.pl (J.M.); dorota.nowak@uwr.edu.pl (D.N.); 2 Department of Oncology and Division of Surgical Oncology, Wroclaw Medical University, Plac Hirszfelda 12, 53-413 Wroclaw, Poland; zietek.m@dco.com.pl (M.Z.); rafal.matkowski@umed.wroc.pl (R.M.); 3 Wroclaw Comprehensive Cancer Center, Plac Hirszfelda 12, 53-413 Wroclaw, Poland

**Keywords:** immune cells, melanoma, targeted therapies, drugs resistance

## Abstract

The low efficiency of currently-used anti-cancer therapies poses a serious challenge, especially in the case of malignant melanoma, a cancer characterized by elevated invasiveness and relatively high mortality rate. The role of the tumor microenvironment in the progression of melanoma and its acquisition of resistance to treatment seems to be the main focus of recent studies. One of the factors that, in normal conditions, aids the organism in its fight against the cancer and, following the malignant transformation, adapts to facilitate the development of the tumor is the immune system. A variety of cell types, i.e., T and B lymphocytes, macrophages, and dendritic and natural killer cells, as well as neutrophils, support the growth and invasiveness of melanoma cells, utilizing a plethora of mechanisms, including secretion of pro-inflammatory molecules, induction of inhibitory receptors expression, or depletion of essential nutrients. This review provides a comprehensive summary of the processes regulated by tumor-associated cells that promote the immune escape of melanoma cells. The described mechanisms offer potential new targets for anti-cancer treatment and should be further studied to improve currently-employed therapies.

## 1. Introduction

Until recently, melanoma constituted only 4% of all dermatological cancers; however, it was responsible for 80% of skin-cancer-related deaths [1]. The standard of care is the surgical removal of the primary tumor and localized metastases; nevertheless, systemic treatment is widely used [2]. Unfortunately, its effectiveness is relatively low, with high toxicity and rapidly emerging resistance to the administered drugs. To minimize the side effects of systemic treatment and eliminate all tumor cells as efficiently as possible, it seems necessary to develop a therapy directed against molecules specific for each type of cancer.

In melanoma, the *BRAF* V600E gene mutation seems to be particularly interesting considering its presence is detected in about 40–50% of patients [3,4,5]. Following the substitution mutation in the *BRAF* gene, in the amino acid sequence, valine is replaced with glutamic acid at position 600 (BRAF V600E) of the polypeptide chain, which results in a constitutively active kinase [6]. To date, monotherapies using small-molecule inhibitors of BRAF V600E (e.g., vemurafenib and dabrafenib) have been approved for clinical use in patients with inoperable and metastatic melanoma, followed by the introduction of the BRAF/MEK (mitogen-activated protein kinase kinase) combination treatment, owing to the quickly emerging resistance based on the reactivation of the mitogen-activated protein kinase (MAPK) pathway in patients treated with single-agent therapy [7,8].

Unfortunately, even a dual therapeutic strategy may lead to the appearance of resistance driven by a variety of mechanisms. It may be associated with the occurrence of subsequent mutations within signaling pathways’ related genes or as a result of adaptive melanoma cell plasticity, which is characterized by transcriptionally distinct phenotypes responsible for a vast intra- and intertumoral heterogeneity of this cancer. Malignant cells can display a more ‘proliferative’ or ‘invasive’ phenotype defined by their transcriptional master regulators—microphthalmia-associated transcription factor (MITF) and AXL, respectively. This phenomenon is greatly dependent on the interaction of melanoma cells with the surrounding tumor microenvironment (TME), which is also highly involved in the development of therapy resistance. In the tumor niche, many types of cells are present, among others cancer-associated fibroblasts (CAFs), keratinocytes, adipocytes, and immune cells. The extracellular matrix that fills the space between the cells and the molecules secreted by neighboring cells may also influence the effectiveness of the treatment. 

In this review, we will focus on the role of immune cells that could recognize and subsequently eliminate cancer cells, though only if they work properly. The above-mentioned cells residing in the tumor niche also contribute to the immune escape of melanoma and will be described further in this particular context. Because melanoma is one of the most immunogenic tumors, associated with the formation of a large number of neo-antigens occurring as a result of chromosomal rearrangements or genetic polymorphisms, it has the highest potential to elicit a specific anti-cancer immune response [9]. For this reason, immune cells are the target of modern anti-melanoma therapy, directed mainly against programmed cell death protein 1 (PD-1) and cytotoxic T-lymphocyte associated protein 4 (CTLA-4). Unfortunately, as in the case of BRAF and MEK inhibitors, melanoma patients sometimes do not respond or become resistant to this form of treatment. 

This review summarizes the current knowledge concerning the functioning of the immune system during melanoma progression and related therapeutic goals that are or could potentially be used as targets in melanoma treatment. 

## 2. Immune Cells Present within the Melanoma Microenvironment

### 2.1. Functions of Immune Cells 

Within the tumor niche, numerous immune cells are present, including T lymphocyte subpopulations, B lymphocytes, natural killer cells (NK), dendritic cells (DC), M1 and M2 type macrophages, and immature cells of myeloid origin called myeloid-derived suppressor cells (MDSC) [10]. During the first stages of tumor development, immune cells fulfill their proper function (summarized in Table 1)—they exert anti-cancer effects through induction of transformed cells’ apoptosis, production of anti-tumor cytokines, or cytotoxic reactions. Active NK cells participate in the recruitment of antigen-presenting cells (APCs) by the secretion of cytokines, while macrophages, neutrophils and dendritic cells residing in the tumor niche phagocytize dead melanoma cells and present cancer antigens that activate secondary adaptive immune responses based on T cells [10,11]. 

There are three main types of T lymphocytes: effector (or cytotoxic), helper, and regulatory cells. CD8+ T effector (Teff) cells can recognize antigens presented by immune cells via major histocompatibility complex (MHC) class I molecules and function by inducing a cytotoxic effect in tumor cells. Primed and activated CD8+ cytotoxic T lymphocytes (Tc) act on melanoma cells directly, by releasing perforin and granzyme B, which induce apoptosis, or indirectly, through the secretion of cytokines, including interferon-γ (IFN-γ) and tumor necrosis factor (TNF), which leads to the consecutive appearance of antigens and the expansion of T cells [10,12,13,14]. CD4+ T helper (Th) cells bind to APCs via MHC class II protein complex and, thanks to the cytokines present in the TME, can differentiate into several types of immune cells with distinct roles in the immune response [15]. The activation and expansion of these cell types is necessary for long-term melanoma remission. 

**Table 1:** Anti-melanoma immune system response.

Type of Immune Cell	Role in the Anti-Cancer Response
Natural killer cells	By binding to the tumor cells and releasing cytolytic molecules, they cause tumor cell death. They also participate in the recruitment of APCs by the secretion of cytokines [10].
Macrophages, neutrophils, dendritic cells	Phagocytosis of dead melanoma cells and presentation of cancer antigens that activate secondary adaptive immune responses [10,11].
Th (helper) cells	Binding to the APCs via MHC class II protein complex and secretion of cytokines, eventually leading to tumor cell death [15].
Teff/Tc (effector/cytotoxic) cells	Recognition of antigens presented by immune cells via MHC class I molecules and induction of a cytotoxic effect in tumor cells [10,13].
Treg (regulatory) cells	Secretion of cytokines and chemokines with immunosuppressive activity [16,17].

Abbreviations: APC—antigen-presenting cells; MHC—major histocompatibility complex.

However, for the anti-tumor response of T helper and T effector cells to be triggered in the first place, they must be fully activated via two co-stimulatory signals. The first one is the interaction of the T lymphocyte receptor (TCR) with the MHC molecule, which is dependent on dendritic cells or antigen presentation by cancer cells. In the presence of only one signal, T cells are not fully stimulated and become anergic. This condition is characterized by a lack of interleukin-2 (IL-2) production and cell proliferation in response to the antigen, and can even lead to cell death. Complementary signal involves the binding of co-stimulatory molecules present on T cells (e.g., CD28) to their related receptors localized on APCs (e.g., CD80 and CD86) [18,19].

### 2.2. T Lymphocytes and T Cell-Related Immunotherapy

Unfortunately, described processes are often disturbed following the escape of tumor cells from the control of the immune system. The high plasticity of tumor cells results in the acquisition of mutations, which allows the population of cells to avoid immunity [10]. Melanoma cells evade recognition by T cells thanks to the reduction in tumor-associated antigen expression, aberrations in their processing system, and a decrease in the MHC molecules level, which renders them almost “invisible” to immune cells. 

#### 2.2.1. CTLA-4 and PD-1/PD-L1

In addition to the above-described processes, inhibitory receptors and ligands (e.g., CTLA-4, PD-1, PD-L1 (ligand of PD-1), B7-H2, and B7-H3) negatively stimulate T cells, leading to their anergy. Melanoma can block T cell activation using varied mechanisms, including the increased production of inhibitory molecules such as PD-L1 (ligand for PD-1 receptor). If T cells simultaneously overexpress PD-1, constant exposure to the tumor antigens provokes their immune unresponsiveness. Dysfunctional T lymphocytes lose their effector function while maintaining proliferative abilities [10,20,21]. Interaction between PD-1 and PD-L1 also induces tumor-infiltrating lymphocytes’ (TILs) apoptosis and stimulates differentiation of CD4+ lymphocytes into regulatory T cells (Tregs). The latter conventional task is to inhibit the response from the immune system in order to maintain self-tolerance. Cancer cells exploit their immunosuppressive abilities [22]. It was shown that melanoma growth and progression is correlated with the presence of Treg cells within the tumor and that their recruitment by cancer cells helps them avoid the immune response, mainly through secreted cytokines and chemokines with immunosuppressive activity, such as tumor growth factor β (TGF-β) and interleukins (ILs): IL-10 and IL-35 [15,23,24]. Additionally, the Teff/Treg ratio in the tumor has a predictive value in immunotherapeutic response [16,17,25]. 

**Figure 1:**
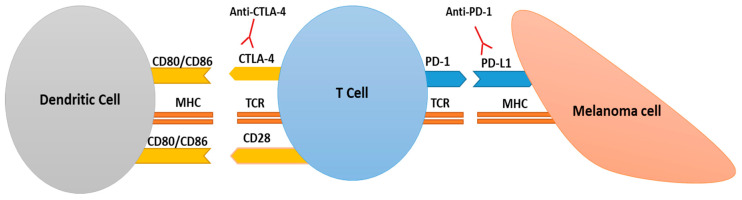
CTLA-4- and PD-1-based immunosuppression. Full activation of the T cell requires the interaction between the T lymphocyte receptor (TCR) and the peptide–MHC complex present on the dendritic or cancer cell as well as the binding of co-stimulatory molecules found on T cells (e.g., CD28) to their receptors localized on dendritic cells (DCs) (e.g., CD80 and CD86). CTLA-4 competes with the CD28 receptor for binding to CD80/CD86 molecules and negatively stimulates these cells, leading to their inactivation. PD-1 binds to PD-L1 found on the surface of tumor cell and, thus, inhibits T cell activity. DC—dendritic cell; CTLA-4— cytotoxic T-lymphocyte associated protein 4; PD-1— programmed cell death protein 1; TCR—T cell receptor; MHC—major histocompatibility complex; PD-L1— ligand of programmed cell death protein 1.

The expression of CTLA-4 molecules on T cells is also often increased in melanoma and, as was mentioned before, may lead to T lymphocyte anergy. This marker competes with the CD28 receptor on the T cell surface for binding to CD80 or CD86 molecules present on the APC. CTLA-4 binds with much higher affinity than CD28 to co-stimulatory molecules, which results in T cell inactivation; for this reason, it is connected with the suppressive phenotype of the TME [19,24]. Furthermore, CTLA-4 is also present on Tregs. Due to the role that CTLA-4 and PD-1 play in the escape of cancer cells from immune system surveillance, they became targets in melanoma immunotherapy (Figure 1).

Anti-PD-1- and anti-CTLA-4-directed drugs belong to the group of immune checkpoint inhibitors (ICI) consisting of monoclonal antibodies that bind to proteins taking part in signaling, leading to T cell anergy (see Table 2) [22,26]. Ipilimumab, accepted by the FDA in 2011 for the treatment of melanoma, blocks inhibitory signaling based on CTLA-4 and, thus, increases the activation and expansion of cytotoxic T cells [27]. 

PD-1 checkpoint receptor is expressed mainly on T cells but partially also on B lymphocytes and NK cells. Therefore, its blockade may potentially inhibit all of these types of cells. The first FDA-approved anti-PD-1 antibody used to treat metastatic melanoma was nivolumab. It binds to PD-1 thereby preventing interaction between the inhibitory receptor and its ligands present in the tumor niche, resulting in greater activity of immune cells. Another anti-PD-1 antibody, pembrolizumab, was certified by the FDA in 2015 for advanced melanoma treatment [28]. When compared, nivolumab treatment increased the median progression-free survival (PFS) to 6.9 months in relation to 2.9 months for ipilimumab and 2.2 for chemotherapy [28]. 

**Table 2:** Immune checkpoint inhibitors used in melanoma treatment.

Drug Name	Therapeutic Target and Effect
Ipilimumab	Human monoclonal IgG1 anti-CTLA-4 antibody blocking inhibitory signaling based on CTLA-4 [27].
Nivolumab	Human monoclonal IgG4 anti-PD-1 antibody binding to PD-1, thereby preventing interaction between this receptor and its ligands present in the tumor niche [28].
Pembrolizumab	Fully humanized monoclonal IgG4 antibody directed against PD-1, which prevents it from interacting with PD-L1 [28].
Atezolizumab	Fully humanized monoclonal IgG1 antibody, interfering with the binding of PD-L1 ligand to its receptors, PD-1 and B7.1 [29].

Abbreviations: CTLA-4— cytotoxic T-lymphocyte associated protein 4; PD-1—programmed cell death protein 1; PD-L1—programmed cell death-ligand 1.

Because CTLA-4 and PD-1 receptors exhibit a different mechanism of action, their combination was tested in clinical trials. The obtained results showed that the median PFS for melanoma patients treated with a nivolumab/ipilimumab mixture was 11.5 months, which is a much better result compared to monotherapies [30]. This combination was recently approved by the FDA for the treatment of advanced BRAF-negative melanoma [22]. Unfortunately, only 50% of patients respond to this method of treatment, and what is more, some of them develop therapy resistance [31]. These ICI-nonresponsive melanoma cells are characterized by the repression of genes that control antigen presentation and IFN-γ signaling [32]. Several reports suggest that the therapeutic success of ICI is more often achieved in the case of a pre-existing tumor lymphocyte infiltration within the tumor microenvironment, which is also associated with a better prognosis for patients [33]. One of the factors with a negative impact on the effectiveness of this form of treatment could be the activation of the WNT/β-catenin signaling pathway, which is positively correlated with the lack of T cell infiltration in melanoma [34]. 

#### 2.2.2. BRAF V600E-Mutated Melanoma

Due to the fact that, according to the preclinical data, BRAF and MEK inhibitors cause changes in the TME and its immunogenicity, an attempt was made to use a combination of anti-MEK/BRAF drugs and immunotherapy. A tumor containing BRAF-mutated melanoma cells exhibits low T cell infiltration and an upregulated level of proinflammatory cytokines, such as IL-6, IL-10, and vascular endothelial growth factor (VEGF), which leads to an increase in the number of immunosuppressive cells, such as Tregs or myeloid-derived suppressor cells, within the tumor microenvironment. 

Moreover, cancer cells with BRAF mutation inhibit the maturation of dendritic cells and, thus, the production of TNF-α and IL-12. The recognition of these cells by the immune system is also reduced due to the low expression of melanoma differentiation antigens (MDA) and a decrease in the level of MHC molecules (see Table 3). This phenomenon may be potentially overcome by anti-BRAF/MEK therapy [35,36,37,38]. Studies based on both melanoma cell lines and a mouse model have shown that treatment of BRAF-mutated melanoma cells with monotherapy directed against BRAF increased T cell infiltration into tumors and upregulated the expression of MDA and MHC class I/II (in the last case, by increasing IFN-γ levels) [39,40]. 

Additionally, in mouse melanoma models, BRAF inhibitors reduced the number of regulatory T cells and MDSCs, with a simultaneous increase in the activity of antigen-presenting DCs, while anti-MEK therapy decreased the mortality of overstimulated effector T lymphocytes [41,42,43]. Furthermore, in the sera collected from patients treated with BRAF inhibitor alone or with a combination of anti-BRAF/MEK drugs, an increased level of immunostimulatory cytokines (IFN-γ, TNF-α, and CCL4) as well as a reduced concentration of immunosuppressive cytokine (IL-8) were detected [36,44].

**Table 3:** Immunosuppressive mechanisms exhibited by BRAF V600E-mutated melanoma.

Immunosuppression in the BRAF V600E-Positive Melanoma
Low T cell infiltration into the tumor [37]
Increase in the number of immunosuppressive cells—Tregs and MDSCs within the TME [35]
Inhibition of dendritic cell maturation and, thus, production of TNF-α and IL-12 [35]
Low expression of melanoma differentiation antigens and a decrease in the level of MHC molecules, which results in diminished recognition of melanoma cells by the immune system [38]

Abbreviations: IL-12—interleukin 12; MDSCs—myeloid-derived suppressor cells; MHC—major histocompatibility complex; Tregs—regulatory T cells; TME—tumor microenvironment; TNF-α—tumor necrosis factor α.

Currently, therapies composed of a mixture of BRAF/MEK inhibitors and immune checkpoint inhibitors are undergoing clinical investigation. The combination of dabrafenib (BRAFi), trametinib (MEKi), and murine anti-PD-1 antibody led to an improved tumor response, visible as a decrease in tumor volume as well as an increase in the number of effector and helper T lymphocytes within the tumor niche, compared to monotherapy in mouse melanoma models [45,46]. Very promising results were obtained in a study in which spartalizumab (anti-PD-1 antibody), coupled with dabrafenib and trametinib, was used to treat patients with advanced BRAF mutant melanoma. A complete response occurred in approximately 40% of cases; unfortunately, it was also associated with serious side effects [32,45,47]. Another clinical trial assessing the anti-tumor activity of a mixture including atezolizumab (anti-PD-L1 antibody), cobimetinib (MEKi), and vemurafenib (BRAFi) reported a stable response (ca. 30 months) in 40% of treated patients [48]. This form of therapy has been recently approved for the treatment of BRAF-mutated advanced melanoma [49].

#### 2.2.3. Potential Markers for Multitargeted Immunotherapy 

In addition to PD-1 and CTLA-4, several other immune checkpoint proteins have been intensely studied to find new therapeutic goals. These include the lymphocyte activation gene-3 (LAG-3), T-cell immunoglobulin- and mucin domain-containing molecule 3 (TIM-3), and T cell immunoreceptor with Ig and ITIM domains (TIGIT). All these markers exhibit a high level of expression in TILs and regulatory T cells, which indicates that they are promising targets for immunotherapy [50]. Clinical trials assessing the effectiveness of their inhibition in melanoma are currently underway. 

Another interesting molecule from the immune checkpoint protein group is glucocorticoid-induced tumor-necrosis-factor-receptor-related protein (GITR). It was shown in preclinical mouse tumor models that modulation of GITR/GITR ligand interaction leads to the inhibition of Treg function with simultaneous activation of effector T cells [51]. Guo et al. demonstrated that ubiquitination and degradation of GITR, leading to the inhibition of T cell-mediated cancer cell death, is dependent on the E3 ligase neural precursor cell expressed developmentally down-regulated protein 4 (NEDD4), whose expression is often elevated in metastatic melanoma. These data suggest that blocking the activity of this enzyme could enhance the anti-cancer immune response [52].

There is potential for other therapeutic strategies that, coupled with standard immunotherapy, may improve overall effect. One of them is directed against sphingosine kinase 1 (SK1), which catalyzes the phosphorylation of sphingosine to sphingosine-1-phosphate—a well-known regulator of lymphocyte trafficking and differentiation. High SK1 activity leads to an increased expression of immunosuppressive factors in the TME, regulatory T cell accumulation, and impaired response to anti-CTLA-4 or anti-PD-1 treatment. Imbert et al. showed that SK1 knockdown in melanoma tumors reduced the production of immunosuppressive cytokines (TGF-β, IL10, CCL17, and CCL22) and decreased Tregs tumor infiltration, while its inhibition significantly sensitized melanoma to immune checkpoint inhibitors treatment [53]. 

Receptors present on T lymphocytes’ surfaces may also provide a basis for the development of new anti-melanoma strategies. T lymphocyte receptor-engineered-T cells, that are derived from the patients’ blood then ex vivo genetically modified and expanded, recognize specific tumor antigens and induce an immune response. Following therapy targeting the melanoma antigen recognized by T cells 1 (MART-1), regression was achieved in 30% of the melanoma patients subjected to the clinical trial [54]. Another study involving TCR-transgenic T cells, also directed against MART-1, reported improvement in 70% of the treated metastatic melanoma cases [55]. However, the effectiveness of adoptive T cell therapies could be hindered by the TNF-α-induced reversible loss of melanoma-associated antigens, which promoted melanoma plasticity and led to the appearance of resistant, dedifferentiated cancer cells [56].

#### 2.2.4. Metabolic Mechanisms Involved in Melanoma Immunosuppression

In addition to the processes blocked by currently-used immunotherapy strategies, there are other mechanisms related to T cells used by cancer cells to escape immune system control (summarized in Table 4). One of them is associated with L-arginine metabolism, and in particular with reduced levels of arginosuccinate synthetase in melanoma cells, which renders them unable to produce arginine [57,58,59]. Cancer cells deprive T cells of this amino acid through their high uptake rates, leading to decreased proliferation and survival of immune cells [57,60]. Importantly, L-arginine is also a precursor to the synthesis of nitric oxide (NO), which is a key immunomodulatory factor that exerts suppressive effects for T cell expansion and function [61,62]. 

Another metabolic product that acts immunosuppressively is adenosine obtained from ATP through CD39 and CD73 ectonucleotidase activity. Adenosine has been shown to inhibit NK cell infiltration and function, impair macrophage activation, and promote the maturation of Tregs, while impeding effectory T cell initiation, their proliferation, and release of cytokines [63]. Because tumor-associated myeloid cells, including macrophages, DCs, and MDSCs, express adenosine-binding receptors (AR), they are potential targets of AR blockers, which may enhance immune reactions against tumor cells [63]. 

Another factor augmenting immunosuppression is a protein released by macrophages and myeloid-derived suppressor cells: indoleamine 2,3-dioxygenase (IDO). Physiologically, this enzyme is involved in immune tolerance during pregnancy to prevent fetal rejection. In melanoma, its high level is associated with poor prognosis for patients [64,65]. IDO inhibits the immune response by reducing the level of tryptophan, which is necessary for lymphocyte proliferation. In addition, it promotes Treg migration into the tumor area, inducing an immunosuppressive effect [66]. Local tryptophan deficiency caused by IDO activates the process of autophagy, leading to T cell anergy [67,68,69]. 

IDO also catalyzes the conversion of tryptophan to kinurenine, whose level is increased in the tumor microenvironment, where it directly inhibits natural killer cell cytolytic activity through downregulation of activating receptors (NKp44, NKp30, and natural killer group 2D receptor (NKG2D)) [70]. Moreover, kinurenine binds to the aryl hydrocarbon receptor, activates it, and, thus, promotes regulatory T cell differentiation, APC immunogenicity reduction, and upregulation of PD-1 expression on Teff cells [71,72]. Preclinical data indicate the synergism of IDO and immune checkpoint inhibitors in reducing the growth of the tumor, thereby offering a promising therapeutic strategy for melanoma, especially considering the fact that the use of IDO-blocking agents has not been associated with high toxicity [69,73]. 

Additionally, it was noted that IDO expression is induced by cyclooxygenase-2 (COX-2) activity, which suggests that COX-2 inhibitors may represent a reasonable additive to currently used therapies to block IDO immunosuppressive effects [74,75]. This hypothesis was confirmed by Ferreira et al. who showed that ibuprofen, an inhibitor of prostaglandin E2 and COX-2, which belongs to the non-steroidal anti-inflammatory drugs (NSAIDs) group, strongly synergizes with PD-1 therapy in a treatment of murine model of melanoma [76]. This suggests that well-known NSAIDs may enhance the effectiveness of anti-PD-1 treatment in melanoma patients who initially did not respond to a PD-1-targeted monotherapy.

**Table 4:** Immunosuppressive factors used by cancer cells to escape immune system control.

Immunosuppressive Factor	Action
Decreased production of arginine	Arginine-deprived T cells exhibit decreased proliferation and survival [57,60] L-arginine is a precursor of nitric oxide synthesis, which inhibits T cell expansion and function [61,62]
High level of adenosine	Inhibition of NK cells infiltration and function [63] Impairment of macrophage activation [63] Promotion of the Treg maturation [63] Impediment of Teff initiation, proliferation, and ability to release cytokines [63]
High level of indoleamine 2,3-dioxygenase (IDO)	Inhibition of the immune response by reduction in the tryptophan level, necessary for T cell proliferation [66] Promotion of Treg migration into the tumor area [66] IDO-mediated local tryptophan deficiency activates the process of autophagy and leads to T cell anergy [68,69]
High level of kinurenine	Inhibition of NK cell cytolytic activity [70] Promotion of Treg differentiation, reduction in APC immunogenicity, and upregulation of PD-1 expression on Teff cells [71,72]
Acidification within the tumor niche	Stimulation of TAMs polarization towards M2 type [77] Reduction in CD8+ T cells cytolytic activity [78] Increased secretion of IL-1β by monocytes and TAMs [78]
Exosomes	Source of ligands, such as PD-L1, which inhibit the anti-tumor response through interaction with receptors present on T cells [79] Transport of soluble factors, such as Fas and TRAIL, which induce Teff cell apoptosis [80] Exosomes with high IL-6 content can inhibit monocyte differentiation into DCs [81]

Abbreviations: NK—natural killer cell, Tregs—regulatory T cells, Teffs—effector T cells, NKG2D—natural killer group 2D receptor, IL-1β—interleukin 1β, PD-1—programmed cell death protein 1, TAMs—tumor-associated macrophages, PD-L1—programmed death ligand 1, CTLA-4— cytotoxic T-lymphocyte associated protein 4, DCs—dendritic cells, TRAIL—TNF-related apoptosis inducing ligand, IL-6 - interleukin 6.

## 3. B Lymphocytes

The majority of the data described above relate to T lymphocytes. However, other types of cells also support cancer progression and may constitute a potential target for anti-melanoma therapy (data for T lymphocytes and other immune cell types are summarized in Table 5). Current knowledge concerning B lymphocytes’ involvement in the tumor development is limited, and in the case of melanoma, the role of B cells is controversial. 

Ladanyi et al. have observed a higher density of B lymphocytes in inflammatory infiltration into the non-metastatic melanoma or a tumor exhibiting invasion only to the lymph nodes, compared to cases in which visceral metastases occurred. Hence, in this case, high B cell content was associated with a better prognosis [82]. Differing results were obtained by Somasundaram et al. They observed that human melanoma cells produce fibroblast growth factor 2, which stimulates B cells that infiltrate the tumor to produce insulin-like growth factor 1 (IGF-1). This factor is crucial for melanoma resistance to BRAF and MEK inhibitors, which is associated with increased levels of CD20 and IGF-1 mRNA in tumors and IGF-1 expression in tumor-associated B lymphocytes. Moreover, the first clinical data from a pilot study of patients with drug-resistant metastatic melanoma showed anti-tumor activity of the anti-CD20 antibody that led to B cell depletion [83]. Potential treatment could comprise of combination of B cell targeted drugs and established kinase or immune checkpoint inhibitors.

## 4. Tumor-Associated Macrophages

Tumor-associated macrophages (TAMs) are present inside the tumor or in the peritumoral region [84]. They are recruited by chemokine CCL2 released by the cancer cells or stroma [85]. TAMs are particularly involved in extracellular matrix degradation, tumor cell migration and angiogenesis. An increase in the number of TAMs in inflammatory infiltration may be a reliable prognostic marker [86]. 

There are two types of TAMs: M1 macrophages, which exert anti-tumor effects, and M2 macrophages, which participate in tumor progression and invasion and are present in advanced stages of cancer [87]. Falleni et al. examined skin melanoma samples at all stages of cancer development and found that M2 macrophages are predominantly present in inflammatory infiltration from an early stage, while M1 macrophages with anti-cancer activity are found intratumoral in low numbers [86]. M2 cells may also downregulate M1-mediated functions. 

M1 macrophages are generally activated by Th1 cells and pro-inflammatory factors, e.g., lipopolysaccharide, IFN-γ, and granulocyte-macrophage colony stimulating factor (GM-CSF), while M2 cells are activated by Th2 cells and anti-inflammatory stimuli, such as IL-4, -10, or -13, or monocyte colony-stimulating factor. It is not entirely clear why the balance between M1 and M2 shifts in tumors toward M2 type; however, it seems that the lack of proinflammatory molecules or the increased presence of Th2 lymphocytes in the tumor may promote a change in the phenotype of macrophages [85,88,89]. IL-10, a compound stimulating the transition of macrophages from the M1 to the M2 type, downregulates MHC class II antigens and reduces the expression of CD80 present on macrophages, which then inhibits cytokine production by Th1 cells [90,91]. 

In melanoma, TAMs are also involved in angiogenesis through the regulation of VEGF-A and IL-8 secretion [85,92,93]. Roda et al. reported that macrophages stimulated with GM-CSF at a low oxygen concentration secrete a high level of a soluble form of the vascular endothelial growth factor receptor 1 (sVEGFR-1), which neutralizes VEGF and inhibits its biological activity; this suggests that administration of GM-CSF might reduce tumor growth and angiogenesis in patients with melanoma through the induction of sVEGFR-1 expression [94]. Additionally, Chen et al. showed that TAMs secrete adrenomedullin, a compound dilating blood vessels, leading to the increased angiogenesis and growth of melanoma cells as well as induction of the polarization of macrophages toward the M2 phenotype. Anti-adrenomedullin-directed antibody and an antagonist of its receptor reduced TAM-induced angiogenesis in vitro and melanoma growth in vivo [95]. 

In addition, tumor-associated macrophages secrete several cancer-stimulating molecules, such as IL-1, TNF-α, IFN-γ, angiotensin, COX-2, and IL-1β, to support the growth, angiogenesis, and metastasis of melanoma [96,97]. Melanoma cells exhibit a high activation level of the IL-1 β/COX-1 axis, which leads to the upregulation of tumor-promoting factors (i.e., IL-1β, IL-8, and VEGF) expression by macrophages, as well as their phenotype switch toward the M2 type. Therefore, inhibition of IL-1β may help in the reactivation of specific antitumor immunity and in a therapeutic M2 to M1 macrophage switch, while administration of COX-2 inhibitors (e.g., aspirin) may aid in overcoming the mechanisms of cancer immunosuppression [98]. 

Moreover, Smith et al. indicated that macrophages reduce the susceptibility of melanoma cells to apoptosis induced by MEK inhibitors in a TNF-α- and MITF-dependent manner [99]. On the other hand, under the influence of various stimuli (e.g., anti-cancer drugs) melanoma cells develop a senescent, MITF^LOW^ phenotype, which is accompanied by specific, senescent-associated secretome. One of the cytokines produced by these slowly proliferating cells is TAM-recruiting CCL2, in which neutralization with antibodies obstructs its pro-invasive abilities [85,100].

## 5. Myeloid-Derived Suppressor Cells 

Tumor growth is also associated with changes in myelopoiesis and the recruitment of immunosuppressive cells, called MDSCs. This heterogeneous population of immature myeloid cells constitutes the precursors of dendritic cells (DCs), macrophages, and granulocytes [101]. Their expansion and migration can be induced by molecules produced during chronic inflammation and cancer, such as GM-CSF, IL-6, IL-10, IFN-γ, and VEGF [102]. In melanoma, C-C chemokine receptor type 5 (CCR5) ligands (i.e., CCL3, CCL4, and CCL5) appear to play a major role in this process [10]. Administration of CCR5-Ig fusion protein to mice with CCR5+ MDSCs resulted in inhibition of melanoma growth, which was associated with reduced immunosuppressive potential of myeloid-derived suppressor cells in tumor [103]. 

The inhibitory effect of myeloid cells on antitumor immunity is associated with high PD-L1 expression, IDO synthesis leading to cytotoxic T cell anergy, as well as IL-10 and TGF-β secretion, which inhibit T cell trafficking [102]. At the same time, MDSCs can stimulate Treg activity [101]. Moreover, myeloid cells control cytotoxic T cell responses using enzymes involved in arginine metabolism—nitric oxide synthase (NOS), which generates NO, and arginase 1 (Arg1) responsible for the reduction of the arginine level. Induction of one of these enzymes’ activity decreases the rate of T cell proliferation. Simultaneous stimulation of these proteins results in a limited amount of arginine, which, combined with peroxynitrites generated by NOS, leads to apoptosis of activated T lymphocytes [61]. Inhibitors blocking both enzymes could comprise a promising therapeutic strategy for restoring the anti-cancer immune function of T cells. 

Another method of restricting MDSCs’ immunosuppressive activity is a treatment directed against phosphodiesterase 5, whose inhibitors downregulate Arg 1 and NOS expression [104]. One of them, tadalafil, has been used among patients with metastatic melanoma. It was well tolerated and 25% of patients showed disease stabilization [105]. 

Under the influence of tumor-released factors, MDSCs are also able to differentiate into endothelial-like cells [106]. Moreover, myeloid cells present in melanoma patients produce several factors (e.g., IL-8, matrix metalloproteinases—MMP-8/9, and VEGF) promoting angiogenesis [85]. In addition, an abnormal increase in the level of STAT3, a regulator of MDSCs’ development, was detected in the myeloid cells of patients with metastatic melanoma. Poschke et al. showed that inhibition of this transcription factor almost completely abolished the suppressive effect of myeloid-derived suppressor cells [107]. 

Another promising treatment combination comprises all-trans retinoic acid and ipilimumab. This dual therapeutic strategy decreases the frequency of circulating MDSCs and reduces the expression of PD-L1, IL-10, and IDO, as well as increases the number of activated CD8+ T cells compared to ipilimumab monotherapy [108]. Furthermore, epigenetic modulation induced by etinostat (an inhibitor of histone deacetylase) was able to strengthen the antigen presentation in tumor cells and block the immunosuppressive activity of MDSCs and Tregs. When entinostat was combined with anti-PD-1 antibodies, the objective response was achieved among 19% of melanoma patients who were originally nonresponsive to anti-PD-1 therapy [32,109]. These data indicate that epigenetics may also be used to overcome drug resistance.

## 6. Dendritic Cells

Another type of immune cell present within the tumor microenvironment is a dendritic cell. They migrate to the lymph nodes in order to present antigens to naive T cells. Roberts et al. showed that in melanoma, DCs bearing CD141 assist in intratumoral CD8+ T cell activation. To exert this effect, dendritic cells must exhibit expression of the CCR7 receptor. Loss of this protein resulted in defects in T cell activation and increased cancer growth, while a high CCR7 level in tumors was related to better clinical outcomes [110]. Moreover, Tucci et al. noted that the number of dendritic cells was lower in patients with metastatic melanoma as compared to those without metastases and inversely correlated with the amount of Tregs. A positive correlation was also observed between the number of dendritic cells and a low risk of recurrence [111]. 

On the other hand, there are some reports indicating that DCs can promote regulatory T cell responses and Teff anergy. Di Blasio et al., using a human organotypic skin melanoma culture, showed that cancer cells induced a phenotype switching of human, naturally-occurring, mature dendritic cells into DCs’ CD14+ variant. These specific cells express COX-2 or IL-6, which are related to immunosuppressive activity, and display a poor stimulatory ability toward effector T lymphocytes [112]. It was also shown that immature DCs may possess immunosuppressive activity while mature dendritic cells can stimulate T cell immune responses [113]. Furthermore, melanoma cells impair DCs’ recruitment and maturation through VEGF and TGF-β secretion and, thus, impede cancer cell targeting by T cells [10,114]. 

It was reported that the Wnt/β-catenin pathway activated by human melanoma cells exerts an immunosuppressive effect on DCs and cytotoxic T cells, mainly through the induction of IL-10 secretion [115]. On the other hand, a melanoma-derived Wnt5a ligand decreases the expression of MITF, which leads to downregulation of the melanoma-associated antigen level and promotes a more “migratory” phenotype. Therefore, this ligand could become a promising new therapy target able to enhance the effectiveness of immunotherapy through the induction of cytotoxic T cells [116]. 

Moreover, under the influence of Wnt5a, DCs upregulate the expression and activity of IDO and, thus, drive Treg differentiation, which serves to repress melanoma immune surveillance. The pharmacologic inhibition of protein-serine O-palmitoleoyltransferase porcupine (PORCN), an enzyme necessary for Wnt ligand secretion, synergistically suppressed melanoma progression when combined with anti–CTLA-4 antibody treatment [117]. Furthermore, Zhao et al. demonstrated that blockage of this pathway enhanced anti-melanoma immunity and increased the activity of anti-PD-1 immunotherapy in a transgenic mouse melanoma model [118]. Immune escape associated with inhibition of CD8+ T cell proliferation may also be facilitated by nitric oxide produced by DC-derived macrophages [119]. 

## 7. Neutrophils

Neutrophils are another type of cell present in the inflammatory infiltrates in melanoma and their numbers gradually increase during tumor progression. Neutrophil mobilization can be achieved by molecules that bind to chemokine receptors, including CXCL1, CXCL2, CXCL3, CXCL5, and CXCL8 [120]. Interestingly, exposure to UVB radiation, which is a major risk factor for melanoma, induces the production of CXCL1 and CXCL8 in the epidermis, thus supporting the recruitment of neutrophils with anti-cancer activity [121,122]. 

As with macrophages, neutrophils also can polarize toward the N1 type with anti-tumor activity or the N2 type, which exhibits immunosuppressive effects. It seems that at the beginning of cancer development, N1 neutrophils predominate in the tumor microenvironment, mediating the killing of melanoma cells, while N2 type cells are, in particular, present in more advanced stages of tumor progression [123,124]. The mechanisms responsible for the switching of neutrophil phenotypes are not fully understood. It has only been shown that circulating tumor cells promote metastasis by secreting G-CSF and CXCL6, followed by recruitment of N2 neutrophils [125]. 

Melanoma utilizes neutrophils to produce IL-8, which has been shown to regulate their mobilization and activity as well as support the extravasation of cancer cells [85,126]. Cancer-associated neutrophils appear to play a role in malignant angiogenesis and invasion, which are processes associated with the ability of these cells to secrete several proteases involved in extracellular matrix remodeling, i.e., MMP-9 [127]. Pro-tumor neutrophils may also contribute to the immune escape by cancer cells through the expression of immune checkpoint proteins (i.e., PD-L1), overexpression of other immunosuppressive molecules, such as IDO and NOS, or secretion of the molecules involved in Treg recruitment (i.e., IL-17) [10,128]. Given the above findings, blockage of reactive neutrophils emerges as a strategy to enhance cancer immunotherapies. 

It was indicated that MET (hepatocyte growth factor receptor), whose high level positively correlates with the more aggressive melanoma phenotype, promotes the activation of signaling pathways leading to the mobilization of neutrophils in response to cancer immunotherapies. This resulted in the acquisition of immunosuppressive properties by these cells as well as the quenching of treatment-induced T cell expansion and effector functions [129,130]. An anti-MET drug impaired a relevant population of neutrophils in the tumor niche, without blocking the total number of neutrophils, and, thus, avoiding higher susceptibility for infections among patients [130]. The above-described data suggest that immunotherapy in combination with anti-MET drugs may elicit a satisfying therapeutic effect. 

## 8. Natural Killer Cells

To further avoid immune system surveillance, melanoma cells are also able to block the functioning of natural killer cells. When NK cells work properly, they recognize tumors that are resistant to T cell killing, thus playing a complementary role in anti-cancer activity. However, melanoma cells reduce the expression of major NK receptors associated with immune function, including NKp30, NKp44, and NKG2D, which leads to the impairment of natural killer cell-mediated cytolytic activity against cancer cells. This inhibitory effect is induced mainly by IDO and PGE2 [70]. In co-culture experiments using cells isolated from patients, it was also shown that cytokines released by the NK cells (e.g., IFN-γ and TNF-α) can induce phenotype switching of melanoma cells toward an undifferentiated, senescent, and more invasive variant characterized by downregulation of MITF and elevated expression of stemness markers [131].

**Table 5:** Role of selected immune cell types in melanoma immunosuppression.

Cell Type	Role in Melanoma Immunosuppression
T lymphocytes	Increased expression of CTLA-4 molecules on T cells negatively stimulates T cells leading to their anergy [10] Elevated production of PD-L1 in melanoma cells combined with the PD-1 overexpression in T cells results in T cell exhaustion [20] Interaction between PD-1 and PD-L1 induces the differentiation of CD4+ lymphocytes into regulatory T cells (Tregs), which secrete cytokines and chemokines with immunosuppressive activity (TGF-β, IL-10, and IL-3) [20,21]
B lymphocytes	Melanoma cells produce FGF2, which stimulates B cells infiltrating the tumor to produce IGF-1—a molecule crucial for melanoma resistance to BRAF and MEK inhibitors [83]
Tumor-associated macrophages (TAMs)	Promotion of angiogenesis through the regulation of secretion of VEGF and IL-8 as well as adrenomedullin [95] Secretion of cancer-stimulating molecules, such as IL-1, TNFα, IFN-γ, angiotensin, COX-2, and IL-1β, to support the growth and metastasis of cancer cells as well as phenotype switch of TAMs toward the M2 type [96,97] Reduction in melanoma cells’ susceptibility to apoptosis induced by MEK inhibitors in a TNF-α- and MITF-dependent manner [99]
Myeloid-derived suppressor cells (MDSCs)	High expression level of PD-L1 and IDO (leading to cytotoxic T cell anergy) as well as IL-10 and TGF-β (inhibition of T cell trafficking) [101,102] Stimulation of regulatory T cell activity [101] Regulation of cytotoxic T cell responses in an arginine-dependent way (nitric oxide synthase and arginase 1) [61] Production of pro-angiogenic factors (e.g., IL-8, MMP-8/9, platelet factor, and VEGF) [85]
Dendritic cells (DCs)	Melanoma-induced phenotype switching of mature DCs into DCs’ CD14+ variant (characterized by COX-2 or IL-6 expression) leads to a reduction in stimulatory ability toward effector T cells [112] Impaired DC recruitment and maturation mediated by VEGF and TGF-β, leading to decreased cancer cell targeting by T cells [10,114] Wnt/β-catenin pathway-dependent suppression of DCs results in IDO upregulation and, thus, promotion of Tregs’ differentiation and immunosuppressive activity [115,132]
Neutrophils	Production of IL-8, which regulates their mobilization and activity, as well as supports the extravasation of cancer cells [85,126] Promotion of malignant angiogenesis and invasion through secretion of proteases involved in ECM remodeling i.e., MMP-9 [127] Expression of PD-L1 and IDO, as well as NOS overexpression, and secretion of the molecules involved in Treg recruitment (i.e., IL-17) [128]
Natural killer cells (NK cells)	IDO- and PGE2-mediated reduction in NK receptors level leading to the impairment of NK cell-mediated cytolytic activity against cancer cells [70] Induction of phenotype switching of melanoma cells toward an undifferentiated and more invasive variant (characterized by downregulation of MITF and elevated expression of stemness markers) driven by cytokines released by the NK cells (e.g., IFN-γ and TNF-α) [131]

Abbreviations: CTLA-4—cytotoxic T-lymphocyte associated protein 4, PD-1—programmed cell death protein 1, PD-L1—programmed death ligand 1, FGF2—fibroblast growth factor 2, IGF1—insulin-like growth factor 1, BRAF—B-Raf Proto-Oncogene, Serine/Threonine Kinase, MEK—mitogen-activated protein kinase kinase, VEGF—vascular endothelial growth factor, IL-8—interleukin 8, IL-6—interleukin 6, IL-1—interleukin 1, TNF-α—tumor necrosis factor α, IFN-γ—interferon-γ, COX-2—cyclooxygenase-2, IL-1β—interleukin 1 β, MITF—microphthalmia-associated transcription factor, ECM—extracellular matrix, TGF- β—tumor growth factor β, MMP-9—matrix metalloproteinase 9, IDO—indoleamine 2,3-dioxygenase, NOS—nitric oxide synthase, NKG2D—natural killer group 2D receptor.

## 9. Other Elements of the TME Regulating Immune Response

In addition to immune cells, extracellular elements, such as miRNAs or exosomes secreted by cells, as well as other types of cells present in the tumor niche, are also involved in regulating the immune response to cancer using a variety of mechanisms.

### 9.1. microRNAs

MicroRNAs are small, non-coding RNAs with a length of 20–25 nucleotides that are involved in attenuating or completely inhibiting protein translation. These molecules are also able to modulate the melanoma immune microenvironment (summarized in Table 6) [133].

Upregulation of the miR-30b/-30d cluster in melanoma cells is associated with a decreased level of GalNAc7 transferase and increased secretion of IL-10, which impairs the recruitment of effector T cells and enhances Treg infiltration [134]. Recently, it has been discovered that the miRNA panel (miRs: -146a, -155, -125b, -100, -125a, -146b, -99b, and let-7e) is involved in extracellular vesicle-mediated conversion of monocytes to MDSCs and resistance to immune checkpoint therapies in patients [135]. Other miRNAs, such as miRs -16-5p, -17-5p, and -20a-5p, were present in greater amounts in the sera of melanoma patients who responded positively to anti-PD-1 therapy in comparison to those who were resistant to this form of treatment, which may suggest that these miRNAs can be utilized as indicators of therapeutic efficiency [136]. 

Melanoma-derived miR-146a supports immune suppression in this type of cancer. The mice lacking its expression had fewer metastases and lived longer. T cells isolated from these mice exhibited a higher level of STAT1 (the miR-146a target) and its downstream effector, IFN-γ, which reduced cell migration and basal metabolic rate and increased PD-L1 levels on the surface of melanoma cells. Combination therapy consisting of an antagonist of miR-146a and anti–PD-1 antibody resulted in enhanced survival of tested mice over anti–PD-1 treatment alone and could be a novel strategy improving anti-tumor response compared to ICI therapy [137]. Gerloff et al. also showed that miR-125b-5p delivered to macrophages by exosomes produced by melanoma induced a tumor-promoting phenotype in these cells through targeting of the lysosomal acid lipase A expression. Additionally, this miRNA enhances the expression of CD80, which binds to the CTLA-4 receptor present on activated T cells, inhibiting their proliferation and function [138,139]. 

Selected miRNAs could also be related to the emergence of drug resistance. Audrito et al. indicated that low effectiveness of therapy based on inhibitors directed against BRAF and MEK proteins is associated with the upregulation of PD-L1 expression in BRAF-mutated melanoma cell lines in an miR-17-5p-dependent way [140,141]. On the other hand, Li and colleagues demonstrated that miR-28 decreases PD-1 expression on T cells [141,142]. 

**Table 6:** Immunosuppressive and immunogenic roles of miRNAs in the melanoma microenvironment.

The Type of Immune Cells	The Immunosuppressive Role of miRNAs	The Name of miRNA
T cells	Impaired effector T cells recruitment, increased IL-10 secretion, and regulatory T lymphocytes infiltration	miR-30b/-30d [134]
	Increased PD-L1 expression in BRAF-mutated melanoma	miR-17-5p [140,141]
Macrophages	Polarization of macrophages toward the M2 type	miR-21, miR-29a, and miR-125b-5p [143,144,145]
	Enhancement of the expression of CD80, which binds to the CTLA-4 receptor present on T cells, inhibiting their proliferation and function	miR-125b-5p [144]
MDSCs	Conversion of monocytes to MDSCs	miRs: -146a, -155, -125b, -100, -125a, -146b, -99b, and let-7e [135]
	MDSC induction in IL-1β^HIGH^ melanoma	miR-155 [145]
	TGF-β1-dependent increased accumulation and activity of MDSCs	miR-494 [146]
NK cells	Diminished recognition of melanoma cells by NK cells through decreased expression of NKG2D ligands	miR- 34a and miR-34c [147]
	**The Immunogenic Role of miRNAs**	**The Name of miRNAs**
T cells	Positive correlation with anti-PD-L1 therapy efficiency	miR-16-5p, miR-17-5p, and miR-20a-5p [136]
	Decreased PD-L1 expression	miR-28 [141,142]
	Elevated production of IFN-γ corresponding with diminished migration rate of melanoma cells	miR-146a [137]
Macrophages	Polarization of macrophages toward the M1 type	miR-155 [148]

Abbreviations: IL-1β—interleukin 1β, IL-10—interleukin 10, IFN-γ—interferon-γ, MDSCs—myeloid-derived suppressor cells, NK—natural killer cell, NKG2D—natural killer group 2D receptor, PD-L1—programmed cell death protein ligand 1, TGF-β1—tumor growth factor β1.

The role of miR-155 is more controversial because it can exert both immunostimulating and immunosuppressive effects. Usually, it is involved in the polarization of macrophages toward the M1 type, which supports anti-cancer activity. However, in tumors with increased IL-1β signaling, it mediates MDSC induction, partially through MITF downregulation [145,148]. In macrophages, colony-stimulating factor 1(CSF1)/ETS2 transcription factor signaling activation induces miR-21 and miR-29a, which target genes involved in M1 polarization, and, as a result, leads to the reprogramming of these cells toward the M2 type [143,144]. Moreover, upregulation of miR-494 in MDSCs in a TGF-β1-dependent way enhances the accumulation and activity of these cells by targeting a phosphatase and tensin homolog (PTEN) and activation of the AKT signaling. Knockdown of miR-494 reduced the activity of MDSCs and inhibited tumor progression [146]. 

Cancer cells express NKG2D ligands, including UL16-binding protein 2 (ULBP2), which bind to the receptor present on NK cells. Their interaction allows cytotoxic lymphocytes to recognize and eliminate melanoma cells. Heinemann et al. showed that miR-34a and -34c target ULBP2 mRNA and lead to its downregulation, thus diminishing tumor cell recognition by NK cells [147]. 

### 9.2. Exosomes

Cargoes, such as miRNA, can be transported by exosomes, small vesicles secreted by cells into the extracellular space, which carry various molecules (e.g., proteins, lipids, and nucleic acids) and hence support the interaction among cancer cells or between the tumor and its microenvironment. Exosomes derived from cancer cells also affect the functioning of the immune system. They can be involved in the expansion of Tregs, thus enhancing immunosuppression [149]. Exosomes secreted by melanoma cells may also provide membrane-bound ligands, such as PD-L1, which inhibit the anti-tumor response through interaction with the related receptors on T cells [79]. 

In addition, exosomes are able to transport soluble factors, such as Fas and TRAIL (a TNF-associated apoptosis-inducing ligand), which induce Teff cell apoptosis [80]. This process can be associated with increased activation of caspases 3, 7, and 9, as well as downregulation of anti-apoptotic proteins, such as Bcl-2, caused by melanoma-derived exosomes containing miRNAs, such as miR-690 [150]. However, in some cases, the exposure of MHC class I and melanoma-associated antigens (MART-1, gp100, tyrosinase) on exosomes may mimic antigen presentation processes, leading to Teff activation [81]. 

The dual nature of melanoma-derived exosomes was also demonstrated in DCs, where vesicles were able to transfer tumor-associated antigens to DCs in order to promote the activation of cytotoxic T cells. They can also inhibit monocyte differentiation into DCs due to the high IL-6 content [81]. Preliminary phase I studies using DC-derived exosomes have been carried out in patients with advanced melanoma and showed that 27% of them achieved a clinical benefit as a result of this therapy [151,152]. Zhu et al. also showed that exosomes produced by NK cells exert cytotoxic effects on melanoma cells and, thus, may be used as a potential immunotherapeutic strategy [153].

### 9.3. Acidification

One of the extracellular factors influencing the immune response to cancer cells is acidification. Lower pH (6.0–7.0) is characteristic of solid tumors, such as melanoma, which display elevated glycolytic activity and a highly inflammatory signature [78]. While many contradictory results are available, the majority of published data indicates the immunosuppressive role of acidosis, which also promotes a more “migratory” phenotype of melanoma cells defined by a MITF^LOW^/AXL^HIGH^ transcriptional signature [154]. Bohn et al. reported that lowered pH was responsible for functional polarization of TAMs toward the M2 type, which supported tumor growth [77]. The others demonstrated its effect a.o. on the diminished cytolytic activity of CD8+ T cells and the increased secretion of IL-1β by monocytes and TAMs (reviewed in [78]). Targeting pH regulators seems to be a rational direction for improvement of anti-cancer therapy. Indeed, it has been shown to enhance the effectiveness of ICI treatment using the melanoma mouse model [155].

### 9.4. Cancer-Associated Fibroblasts

**Figure 2:**
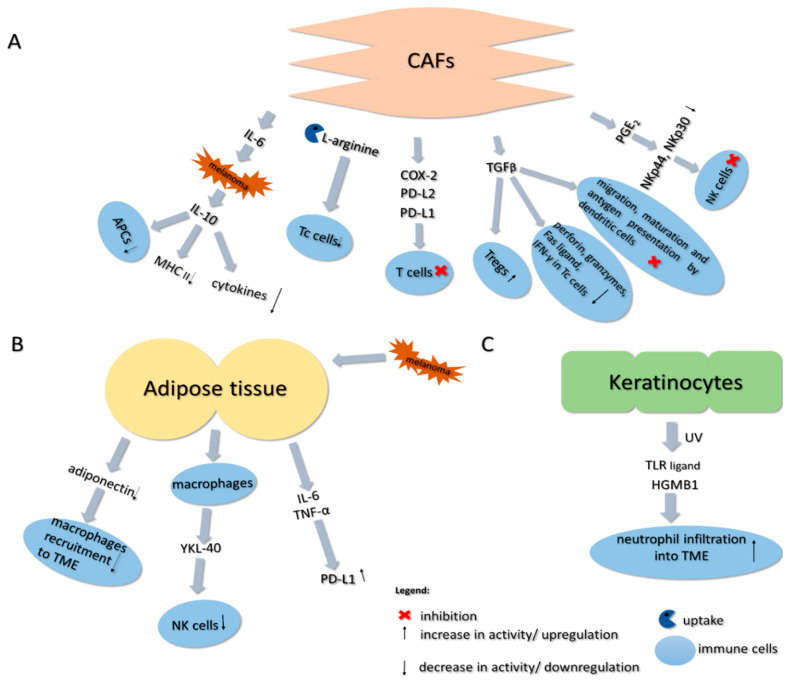
Influence of cancer-associated fibroblasts (**A**), adipose tissue (**B**), and keratinocytes (**C**) on the activity of immune cells present in the melanoma niche. A detailed description is provided in the text. Abbreviations: IL-6—interleukin 6; IL-10—interleukin 10; APCs—antigen-presenting cells; MHC II—major histocompatibility complex type II; Tc cells—cytotoxic T cells; COX-2—cyclooxygenase-2; PD-L2—ligand of programmed cell death protein 2; PD-L1—ligand of programmed cell death protein 1; TGF-β—transforming growth factor β; Tregs—regulatory T cells; IFN-γ—interferon-γ; PGE2—prostaglandin E2; NK cells—natural killer cells; TME—tumor microenvironment; TNF-α—tumor necrosis factor α; UV—ultraviolet; TLR ligand—toll-like receptor ligand; HGMB1—high mobility group box 1.

Fibroblasts present in the tumor vicinity may be converted into cancer-associated fibroblasts (CAFs) that exhibit similar properties to myofibroblasts [156]. CAFs may constitute up to 80% of the tumor mass and display a wide range of functions during the melanoma progression, including an immunosuppressive effect, which, to a large degree, is based on TGF-β secreted by these cells (see Figure 2). This cytokine can inhibit migration, maturation, and antigen presentation by dendritic cells, increase the number of Tregs within the tumor microenvironment, and reduce the expression of perforin, granzymes, Fas ligand, and IFN-γ in cytotoxic T cells [157,158,159,160]. It was also demonstrated that factors expressed by these fibroblasts positively correlate with T cell infiltration and that CAF-rich tumors display an AXL^HIGH^ signature, which is characteristic of the “migratory” phenotype of melanoma. On the other hand, high expression of proliferative marker MITF is associated with the less frequent presence of CAFs in the melanoma TME, which shows an even more complex link between the cancer plasticity and its niche [161]. 

Moreover, it was reported that CAF-targeted therapy enhanced the antitumor activity of cytotoxic T cells and NK cells, simultaneously inhibiting the induction of Tregs and MDSCs [162,163]. Khalili et al. also proved that IL-1α and IL-1β secreted by BRAF V600E-mutated melanoma cells affected the CAFs present in the tumor niche. Following treatment with these cytokines, CAFs overexpressed proteins involved in T cell inhibition, namely, COX-2, PD-L1, and PD-L2, which are known to suppress Teff cells. Neutralization of IL-1α and IL-1β partially reversed the inhibitory effect of CAFs on T cell activity [164]. Furthermore, the accumulation of CAFs within the tumor was associated with low efficacy of PD-1-targeted therapy [165]. CAF-derived MMP-9 is an enzyme responsible for proteolytic PD-L1 cleavage from the surface of melanoma cells and, therefore, leads to anti-PD-1 therapy resistance. *MMP9* knockdown experiments corroborated these findings and showed that CAFs lacking the MMP-9 protease did not affect the PD-L1 level on the melanoma cell surface [32,166]. 

As mentioned above, CAFs secrete TGF-β, which is also involved in the development of the resistance to anti-PD-1 therapy as it contributes to the reduction in MHC class I complex expression in melanoma cells [167]. Ersek et al. reported that melanoma-associated fibroblasts suppress the activity of cytotoxic T lymphocytes and influence Tc signaling via L-arginine depletion. Upon treatment with a CAF-conditioned medium, Tc cells displayed dysregulated ERK1/2 and NF-κB signaling, impeded granzyme B production, and impaired cancer cell killing activity [168]. Moreover, IL-6, which is also secreted by CAFs, induces IL-10 production by melanoma cells. This cytokine, in turn, reduces immune response by suppressing APCs’ function, inhibiting pro-inflammatory cytokine production, and downregulating co-stimulatory molecules and MHC class II expression [169,170]. 

It was also indicated that CAFs may block NK cell activation. These fibroblasts, upon pro-inflammatory factor treatment, inhibit the upregulation of receptors’ (NKp44 and NKp30) expression in a prostaglandin E2-dependent manner, which leads to NK cell inactivation [171]. Furthermore, melanoma-associated fibroblasts reduce the susceptibility of cancer cells to NK-mediated lysis by the secretion of active MMPs, which reduce the amount of NKG2D ligands at the surface of tumor cells and, thus, decrease the NKG2D-dependent cytotoxic activity of NK cells [157].

### 9.5. Adipose Tissue

Adipose tissue (AT) present in the tumor microenvironment also affects the anti-cancer response of immune cells (see Figure 2). It was shown that the β3-adrenergic receptor, located mainly in the adipose tissue, is often upregulated in the melanoma microenvironment, where it promotes tumor growth [172]. Calvani et al. showed that treatment of melanoma-bearing mice with an antagonist of this receptor (SR59230A) promotes active hematopoiesis within the tumor, which can strengthen the immune cells’ anti-tumor response [173]. Wagner et al. demonstrated that peritumoral adipose tissue exhibits a high infiltrate of macrophages closely resembling TAMs, which could also stimulate tumor growth [174]. This effect is even more pronounced in obese individuals, in whom macrophage infiltration induces production of proinflammatory cytokines (CCL2 and MCSF) and angiogenic factors (VEGF) by the tumor [175,176]. On the other hand, Sun and Lodish showed that deficiency of adiponectin, an adipocyte-derived protein, whose level is often downregulated in cancer, promoted the growth of melanoma tumors in mice. It was related to the reduced macrophage recruitment into the TME and could be associated with the inhibitory effects of macrophages on cancer development at the early stages of tumorigenesis [177].

Moreover, Li et al. indicated that obesity in melanoma-bearing mice is positively correlated with the expression of PD-L1, which was associated with the presence of TNF-α and IL-6 released by adipocytes [178]. Furthermore, YKL-40 (chitinase-3-like protein 1), a protein secreted by adipose tissue-derived macrophages, whose level was increased in the sera of obese individuals, was correlated with the poor survival of melanoma patients [179,180,181,182]. This could be partially associated with the fact that YKL-40 was reported to decrease NK cell accumulation and enhance melanoma pulmonary metastasis formation [183]. 

NK cells are dependent on the presence of IL-15, which supports their proliferation and activation as well as induces the survival and activity of Teffs [184,185]. Xiao et al. developed an adipocyte-targeting recombinant adeno-associated viral vector that delivered an IL-15/IL-15Ra complex directly to the mice fat. The subsequent activation of IL-15 signaling resulted in the expansion of the NK cell population, which suppressed tumor growth [186].

### 9.6. Keratinocytes

Keratinocytes are another type of cells abundantly present in the melanoma microenvironment. In normal physiological conditions, the UV-absorbing pigment, melanin, present in the keratinocytes protects melanocytes from mutations induced by long exposure to harmful radiation [187]. However, the UV may also induce cancer progression in a way that is not dependent on tumor-initiating effects. Using a mouse model, Bald et al. showed that prolonged exposure to UV radiation resulted in increased neutrophil infiltration into the melanoma niche that was facilitated by the secretion of Toll-like receptor ligand, high mobility group box 1 (HMGB1), by keratinocytes (see Figure 2). It also promoted the migration of melanoma cells along the external surface of blood vessels and their perivascular invasion. Therefore, inflammatory conditions may induce melanoma plasticity toward a more “migratory” phenotype. Researchers corroborated this data with results obtained from the patients who showed a higher risk of melanoma metastasis that positively correlated with an elevated level of neutrophil infiltration found in the primary tumor, which could be a result of increased HMGB1 secretion [188].

## 10. Conclusions

The melanoma microenvironment is a multi-component and complex network of interactions. The various cells present in the tumor niche contribute to the heterogeneity and plasticity of melanoma, which facilitates its immune escape and the development of therapeutic resistance. It is worth noting that the multitude of cells surrounding the neoplasm (e.g., immune cells, CAFs, adipose tissue, and keratinocytes) can communicate with each other directly or through secreted molecules and, in this way, enhance the immunosuppressive conditions of the tumor niche. In this review, we have described how immune cells and the diverse mechanisms they utilize contribute to the immune escape of melanoma. Based on the recent literature, we have also illustrated the validity of the immunosuppressive processes taking place in the melanoma microenvironment as promising targets for new and adjuvant therapeutic strategies, especially in the case of metastatic cancers resistant to currently used targeted immunotherapies.
